# Scientometric trends and knowledge maps of global health systems research

**DOI:** 10.1186/1478-4505-12-26

**Published:** 2014-06-05

**Authors:** Qiang Yao, Kai Chen, Lan Yao, Peng-hui Lyu, Tian-an Yang, Fei Luo, Shan-quan Chen, Lu-yang He, Zhi-yong Liu

**Affiliations:** 1 School of Medicine and Health Management, Tongji Medical College, Huazhong University of Science and Technology, Wuhan, Hubei 430030, China; 2 Center for Studies of Information Resources, Wuhan University, Wuhan, Hubei 430072, China; 3 Department of Medical Informatics, Biometry and Epidemiology, University of Munich, Ludwig-Maximilians-Universität München, Geschwister-Scholl-Platz München 180539, Germany

**Keywords:** Global trend, Health systems research, Knowledge mapping, Scientometric, Web of Science

## Abstract

**Background:**

In the last few decades, health systems research (HSR) has garnered much attention with a rapid increase in the related literature. This study aims to review and evaluate the global progress in HSR and assess the current quantitative trends.

**Methods:**

Based on data from the Web of Science database, scientometric methods and knowledge visualization techniques were applied to evaluate global scientific production and develop trends of HSR from 1900 to 2012.

**Results:**

HSR has increased rapidly over the past 20 years. Currently, there are 28,787 research articles published in 3,674 journals that are listed in 140 Web of Science subject categories. The research in this field has mainly focused on public, environmental and occupational health (6,178, 21.46%), health care sciences and services (5,840, 20.29%), and general and internal medicine (3,783, 13.14%). The top 10 journals had published 2,969 (10.31%) articles and received 5,229 local citations and 40,271 global citations. The top 20 authors together contributed 628 papers, which accounted for a 2.18% share in the cumulative worldwide publications. The most productive author was McKee, from the London School of Hygiene & Tropical Medicine, with 48 articles. In addition, USA and American institutions ranked the first in health system research productivity, with high citation times, followed by the UK and Canada.

**Conclusions:**

HSR is an interdisciplinary area. Organization for Economic Co-operation and Development countries showed they are the leading nations in HSR. Meanwhile, American and Canadian institutions and the World Health Organization play a dominant role in the production, collaboration, and citation of high quality articles. Moreover, health policy and analysis research, health systems and sub-systems research, healthcare and services research, health, epidemiology and economics of communicable and non-communicable diseases, primary care research, health economics and health costs, and pharmacy of hospital have been identified as the mainstream topics in HSR fields. These findings will provide evidence of the current status and trends in HSR all over the world, as well as clues to the impact of this popular topic; thus, helping scientific researchers and policy makers understand the panorama of HSR and predict the dynamic directions of research.

## Background

With the approach of 2015, many countries intend to hasten their efforts towards meeting the Millennium Development Goals (MDGs), and meanwhile they have already began discussing the post-MDGs health roadmap [1]. In recent years, evidence about the progress towards the MDGs has moved the health systems topic to the center stage, especially in low- and middle-income countries [1,2]. The poor state of health systems in most parts of the developing world is considered one of the greatest barriers for the MDGs to be met, even in some high-income countries such as the US, which has a large percentage of the population without any access to health care due to the inequitable arrangements of social protection. The importance of health systems as part of the global health agenda and in terms of the World Health Organization’s (WHO) response is being reflected in the 11th General Programme of Work (2006–2015) and the Medium-term Strategic Plan (2008–2013). Good health systems not only play a critical important role in improving health, but are widely recognized as being vital elements in the social fabric of every society. They are not only critical for the treatment and prevention of ill-health, but are the central strategies in addressing health inequity and wider social injustice [3]. Well-functioning health systems facilitate the achievement of good health with the efficient use of the available resources. This is achieved by critically increasing the effective responses to the developing public health emergencies by addressing the burden of diseases, ill health, and poverty as a result of communicable and non-communicable diseases and cancers. Effective health systems enable the responsiveness towards legitimizing the expectations of citizens and fairness of financing. By helping in producing effective good health, health systems can also contribute to economic growth [4].

Health systems have existed in some form for over 100 years, when individuals and eventually governments became interested in organizing health systems to protect their population’s health and treat their diseases [5]. However, the concept of health systems is defined in various ways [4]. Generally, health systems can be defined by what they seek to do and achieve, or as the elements by which they are comprised. On the one hand, the WHO defined health systems as “*all organizations, people and actions whose primary intent is to promote, restore or maintain health*” [6]. This definition includes the efforts to address the determinants of health along the direct activities to improve health. Health systems are therefore more than the pyramid of publicly owned facilities that deliver personal health services. On the other hand, definitions of health systems have been based mainly on the utility of achieving health outcomes [7]. The WHO’s building blocks approach is the most popular classification and is widely accepted and used by researchers and decision makers. They conceptualize health systems in the functional or instrumental terms of its constituent “hardware” – service delivery, health workforce, information, medical products, vaccines and technologies, financing, leadership, and governance [5,7]. Therefore, the WHO’s building blocks definition is used herein. Although these building blocks help to clarify the essential functions of health systems, the efforts to address health systems should recognize the interdependence of each part. The building blocks alone do not constitute a system any more than a pile of bricks constituting a functioning building [8]. It is the multiple relationships and interactions among the blocks – how one affects and influences the others and in turn gets affected by them – that convert these blocks into a system [9]. The “software” – by which we mean the ideas and interests, values and norms, and affinities and power that guide actions and underpin the relationships among system actors and elements – are also critical to the overall health systems performance [7,9]. As such, health systems may be understood through the arrangement and interaction of their parts, and how they enable the system to achieve the purpose for which it was designed [5,8].

The current surge in activities and researches around health systems is encouraging. Funding has increased in recent years [10,11] with organizations strengthening health systems such as the Global Alliance for Vaccines and Immunizations and the Global Fund to fight AIDS, tuberculosis, malaria, and other such diseases. Meanwhile, new initiatives have been launched to address some of the bottlenecks to scale up essential health interventions and strengthen some components of health systems [11-13], such as the Implementation Research Platform, the Taskforce on Innovative International Financing for Health Systems, the Evidence-Informed Policy Network, the Canadian-Funded Catalytic Initiative, the Norwegian Government Support to the Results-Based Financing Initiative, the US President’s Emergency Plan for AIDS Relief, and Providing for Health, which is supported by Germany and France. Furthermore, several health systems partnerships have recently emerged, including the Alliance for Health Policy and Systems Research (AHPSR), the Global Health Workforce Alliance, the Health Metrics Network, and the International Health Partnership, among others. In particular, the establishment of the AHPSR in 1999, as a partnership hosted by the WHO, marked an important milestone in the field of health systems [14]. It not only legitimized health systems research (HSR) by demonstrating strong commitment and investment of human and financial resources by the WHO and global funding agencies, but it also provided a platform for international partnership and collaboration and created an identity for this growing field.

Recent studies also have called for intensified investment, methods development, and capacity building in the assessment and research that accompanies health systems investment, ultimately strengthening the implementation processes. AHPSR have published the *Role and Promise of Policy and Systems Research*[15], *Sound Choices: Enhancing Capacity for Evidence Informed Health Policy*[16], *Systems Thinking for Health Systems Strengthening*[17], and most recently *HPSR: A Methodology Reader*[9] in order to strengthen health systems. *PLoS Medicine* commissioned three articles on the state-of-the-art in HSR [7,18,19]. Three Policy Forum articles that were authored by a diverse group of global health academics had critically examined the current challenges of the field and set out what is needed to build up the capacity in HSR to support the local policy development and health systems strengthening, especially in low- and middle-income countries. In addition, during the past decade, a series of conferences and task forces on health research, such as the International Conference on Health Research for Development, Bangkok, 2000, and ministerial meetings in Mexico City in 2004 and Bamako in 2008, had a strong focus on practical and operational questions, which was frequently framed as HSR. The First Global Symposium on HSR, held in Montreux, Switzerland, in November 2010 was the most recent of a succession of conferences and task force deliberations that have spun off a series of debates about the nature of the field and the future directions it should take [7]. The most prominent ones were the launch of the 2010 WHO’s Research for Health Strategy, the Organization of Global Symposia on Health Systems Research in Montreux, Switzerland, and in Beijing, China, and the establishment of a society of health systems researchers known as Global Health Systems. Now, with the launch of the Strategy on HSR on 1 November 2012, the WHO is helping to institutionalize HSR and facilitate evidence-informed decision-making. However, support currently focuses on disease-specific funding for the control of diseases such as HIV/AIDS, tuberculosis, and malaria. Yet, it is increasingly recognized that only limited and short-term gains can be made unless the broader health systems infrastructure is strengthened along large scale interventions that have been introduced [2,20].

Overall, high level meetings and community level advocacy groups have highlighted the challenges that lie ahead: the post-MDGs global health agenda, burgeoning non-communicable diseases, achieving universal health coverage, and strengthening fragile health systems in low- and middle-income countries [21]. Therefore, the consensus on the importance of strong health systems is welcomed. However, because of the importance and high growth rate of HSR both in theory and practice, there have been few attempts or efforts to map global health systems research that is related in context. Without clarity on future directions, focus, and energy this could all dissipate and the global health decision makers could be at a crossroads. Meanwhile, the process of developing an HSR study begins with identifying the topic of focus – the issue or problem you want to investigate – and the related questions. Thus, it is important to identify common trends faced by all health systems, which ultimately allow us to spell out suggestions for reform and change.

The main purpose of this study is to evaluate the global progress and quantitatively assess the current research trends on HSR. A comprehensive scientometric analysis and substantial discussion of research progress in HSR were provided so that specific attempts were employed in order to i) summarize significant publication patterns in HSR with basic statistics as well as advanced analysis, ii) evaluate research performance from multiple perspectives such as year of publication, subject category, journals, countries/regions, and institutes as well as authors [22,23], and iii) present the research foci about international HSR from multiple angles. Moreover, citation data were used as a scientometric tool to indicate the intellectual impact of the research outputs.

## Methods

### Data sources

As a strictly selected abstract database, Web of Science (WoS), including Science Citation Index Expanded, Social Sciences Citation Index, and Arts and Humanities Citation Index, has long been recognized as the most authoritative scientific and technical literature indexing tool providing data on the most important areas in science and technology research, especially about medicine. Furthermore, the WoS database includes the world’s most important journals in relation to healthcare science, health policy, and systems research. The majority of high quality articles on healthcare science research are indexed by WoS. In addition, as a citation database, WoS provides enough search fields, such as keywords, country, organization, author, and references, which are all very important for literature analysis, especially for scientometric analysis [24,25]. Therefore, we conducted a systematic search through the WoS database. The search strategy has been built on previous literature reviews with similar objectives [10,13,26-28], with further refinements and iterative testing of individual search terms. The six building blocks of health systems, as defined by the WHO, were used to define the scope of this search (details of search terms and the search strategy are available in Additional file 1). Literatures were included if they met the following criteria: for the purpose of this study, HSR included the system-level research directly targeting one or more of the six health system building blocks and their sub-components as defined by the WHO. A total of 35,819 publications were identified from 1900 to 2012. The retrieved papers were downloaded from WoS as text files and were converted to a database using the Thomson Data Analyzer, which was also used to clean and analyze data. Papers originating from England, Scotland, Northern Ireland, and Wales were grouped under the United Kingdom heading, while those from Hong Kong, Macao, and Taiwan were categorized each independently and are not included under the China heading.

### Methodologies

Following the best international practices, ‘evaluative scientometrics’ was selected for this study. Scientometrics is a method by which the state of science and technology can be observed through the overall production of scientific literature at a given level of specialization. This tool provides an approach for situating a country in relation to the world, an institution in relation to a country, and individual scientists in relation to their peers. Scientometric indicators are equally suitable for macro-analysis (e.g., a given country’s share in global output of scientific literature over a given period) and micro-studies (e.g., a specified institute’s role in producing articles in a particular field or specialty of science) [25]. In this paper, the distribution of document types, language, year of publication, subjects, journals, countries, institutions, authors, highly multiple cited papers, high frequency keywords, and cluster analysis, as well as collaboration of the subjects, countries, institutions, and authors were thoroughly examined. The total local citation score (TLCS) and the total global citation score (TGCS) were also calculated in this paper. TLCS is the number of times a set of papers included in a collection that has been cited by other papers within the collection. TGCS is the number of times a set of papers included in a collection has been cited in the WoS. The average global citation score (AGCS) is the mean value of TGCS, which also indicates the average citation times of articles in HSR areas. TLCS and TGCS have been the key indicators capable of evaluating the relevance of each research paper in our sample. Meanwhile, an approach considering the average citation per year was also used (TLCS/t and TGCS/t). TLCS/t is the total local citation score per year from the time the research papers’ publication to the end of the sample period, and TGCS/t is the total global citation score per year from the research papers’ publication to the end of the sample period [29]. In addition, the impact factor and H-index are also used to assess the quality of journals. Finally, Thomson Data Analyzer [30], HistCite [31], and VOSviewer [32] software were employed to analyze the publications for knowledge mapping.

The term ‘co-author’ is used to denote the appearance of multiple writers simultaneously in one paper, and also reflects the collaboration of different institutes, regions, or countries [33,34]. The higher the strength of these co-authorships, the closer the relationship among them. Collaboration between countries was determined by the author description, where the term ‘independent’ was assigned if no collaboration was present. ‘Co-words’ refers to the phenomenon that two or more keywords occur simultaneously in one article or one field, where the number of times being cited is called the frequency or strength of co-words [35]. ‘Cluster analysis’ is a collective term covering a wide variety of techniques for delineating natural groups or clusters in data sets [36]. It aims to group a set of objects in such a way that objects in the same group (called a cluster) are more similar (in some sense or another) to each other than to those in other groups (other clusters). During the process, many algorithms and software were used. This study is based on the relationship between countries, institutions, authors, and keywords through a certain algorithm to find the core groups among them by VOSviewer software. It has recently been used in many fields, including machine learning, pattern recognition, image analysis, information retrieval, and bioinformatics [37,38]. Knowledge mapping technology was also referred to as visualization technology, which includes data gathering, survey, exploring, discovery, conversation, disagreement, gap analysis, education, and synthesis. It aims to track the loss and acquisition of information and knowledge, personal and group competencies and proficiencies, show knowledge flows, appreciate the influence of intellectual capital due to staff loss, and assist with team selection and technology matching. Knowledge mapping can not only externalize networks of cognitive relationships and renders them in graphic form, but could also describe a newly evolving interdisciplinary area of science [39]. In this study, co-author, co-word, and cluster analysis methods were used to analyze the collaboration among several research organizations through visualization or knowledge mapping technology [40,41].

## Results and discussion

### Document types

There were 35,819 total HSR-related papers in the Science Citation Index Expanded, Social Sciences Citation Index, and Arts and Humanities Citation Index databases used for this study, distributed over 17 different document types [42]. There were 28,787 research articles comprising 80.37% of the total productions, followed by reviews (2,555; 7.13%), proceedings papers (2,121; 5.92%), editorial material (2,031; 5.67%), meeting abstracts (1,054; 2.94%), letters (531; 1.48%), book reviews (454; 1.27%), and news items (224; 0.63%). Other document types with fewer papers were omitted. Following the conventions used in other scientometric studies, further analysis of articles was restricted, which were peer-reviewed and represent original scientific development. Publications of all other types were thus removed from the analysis of this article.

### Global publication trends

**Figure 1:**
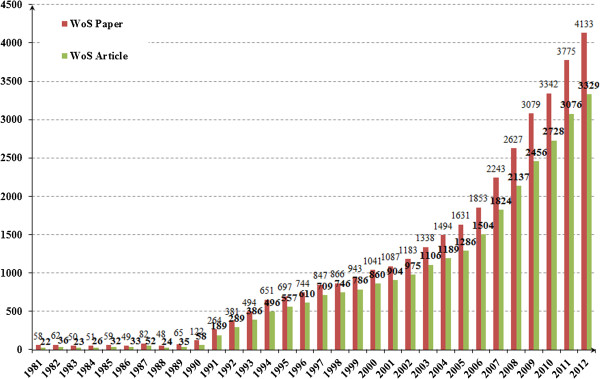
Health systems research-related publications in WoS (1981 to 2012).

The publication trends in annual papers in HSR from 1981 to 2012 are shown in Figure 1, indicating that the developing process of HSR could be divided into three stages in accordance with the growth pattern of literature. The first stage is the infancy period from 1900 to 1990, the second stage is the slow development period from 1991 to 2000, and the third stage is the rapid growth period from 2001 to 2012. During the past decades, WoS papers on HSR that were produced in the 1980s were far less than 100 and mounted up to more than 4,000 in 2012. Meanwhile, WoS articles on HSR exceeded 3,000 papers in 2012. It can be seen from Figure 1 that not many researchers paid attention to the few HSR papers published before 1990, and few proceedings reported the progress of the related work. After 2001, the WoS annual number of publications has grown exponentially, indicating that research has recently garnered more attention (Figure 1).

### Subject categories of publication

Based on the classification of subject categories in the Journal Citation Report of WoS, the publication output data of HSR during the last century was distributed in 140 subject categories. The top 10 productive subject categories are shown in Table 1.

**Table 1:** Top 10 productive subject categories on health systems research

**No.**	**SCI Subject category**	**1900–1990**	**%**	**1991–2000**	**%**	**2001–2012**	**%**	**1900–2012**	**%**
1	Public, Environmental & Occupational Health	157	24.34	1,139	20.24	4,882	21.68	6,178	21.46
2	Health Care Sciences & Services	90	13.95	1,267	22.51	4,483	19.91	5,840	20.29
3	General & Internal Medicine	153	23.72	882	15.67	2,748	12.21	3,783	13.14
4	Psychiatry	54	8.37	420	7.46	1,193	5.30	1,667	5.79
5	Nursing	9	1.40	261	4.64	1,275	5.66	1,545	5.37
6	Pharmacology & Pharmacy	22	3.41	284	5.05	957	4.25	1,263	4.39
7	Psychology	36	5.58	294	5.22	687	3.05	1,017	3.53
8	Biomedical Social Sciences	55	8.53	295	5.24	656	2.91	1,006	3.49
9	Surgery	2	0.31	147	2.61	783	3.48	932	3.24
10	Business & Economics	45	6.98	163	2.90	644	2.86	852	2.96
Total		623	96.59	5,152	91.54	18,308	81.32	24,083	83.66

**Figure 2:**
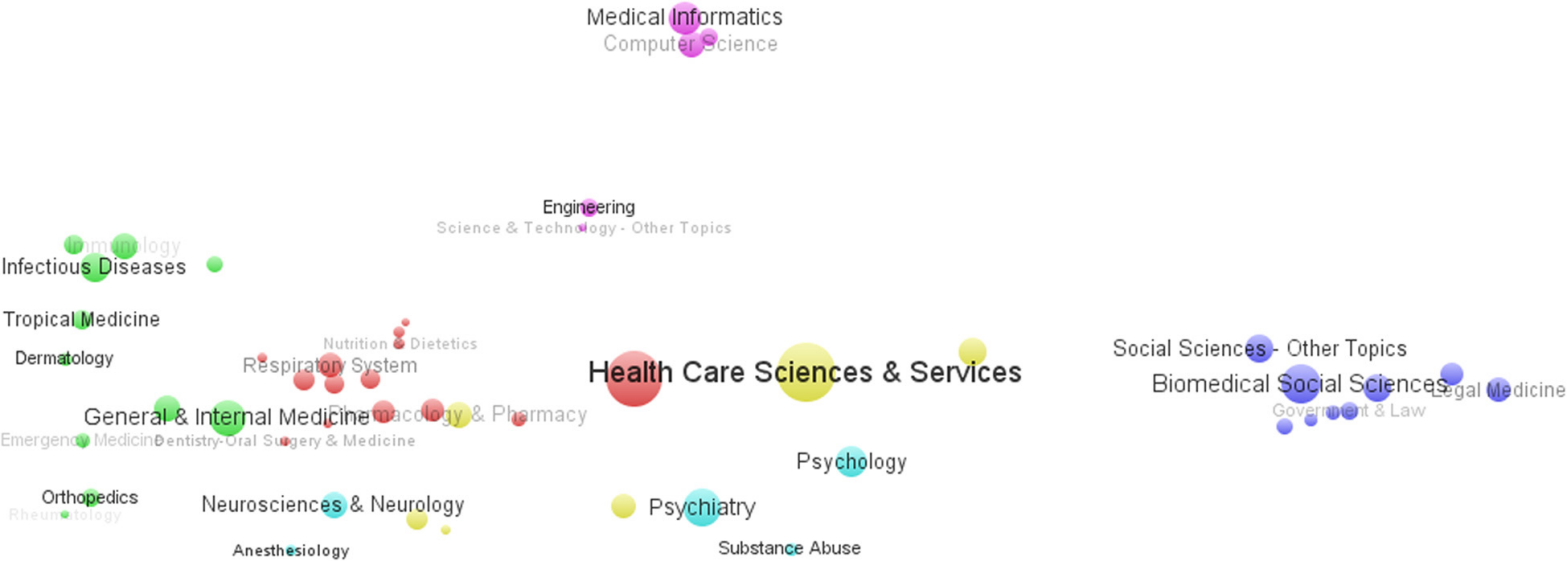
Subjects categories co-occurrence map on health systems research.

HSR was mainly located in the fields of public, environmental and occupational health, health care sciences and services, and general internal medicine as shown in Table 1. Meanwhile, more and more studies have focused on nursing, pharmacology and pharmacy, and surgery. Moreover, psychology, biomedical social sciences, and business and economics also played important roles, where investigators have studied factors and interventions that influence the health of populations. In addition, Table 1 also indicates that research in the above mentioned fields began relatively early. HSR in the subjects of environmental and occupational health and general internal medicine had occupied a dominant position in the earlier stages, while in the past two decades the study of health care sciences and services has gradually exceeded that of general internal medicine. Finally, the number of scientific articles per category has exhibited a trend of rapid growth during the last decade, which indicates that HSR is in a rapid development stage and subsequently needs more efforts (Table 1).To represent the relationship more synthetically between categories, the subjects’ categories co-occurrence network was drawn and visualized in Figure 2. Figure 2 shows that, in the scientific network map of HSR, these subjects are clustered into five subject category groups and shown with different colors. It suggests that HSR is an interdisciplinary area and includes medicine (such as general and internal medicine, surgery, nursing, cardiovascular system and cardiology, infectious diseases, oncology, and other diseases), public-environmental and occupational health, health care sciences and services, pharmacology and pharmacy, economics, sociology (biomedical social sciences, social issues, social work, medical ethics, government and law, public administration), information science and technology (medical informatics, computer science, engineering, information science and library science), and psychology (psychiatry, neurosciences, and neurology). Since health economics is a central discipline of HSR, the analyses most centrally falling within HSR include works that focus on financing (Figure 2).

### Core journals of publication

Thomson Reuters’ WoS covers research published in more than 12,000 scientific journals and conference proceedings, and is presently one of the most extensive sources of research and development outputs. HSR output was published in 3,674 journals, where the top 10 journals with more than 200 articles are displayed in Table 2. These top 10, or 0.27% out of the 3,674 journals, had published 2,969 or 10.31% of the total 28,787 articles and received 5,229 local citations and 40,271 global citations. There was a high concentration on HSR publications in these top journals, where approximately one third of the articles are found in the top 60 most productive journals, a phenomenon that follows Bradford’s law [43] and is consistent with observations in other fields.

**Table 2:** Top 10 most productive journals on health systems research

**No.**	**Journal**	**1900–1990**	**%**	**1991–2000**	**%**	**2001–2012**	**%**	**1900–2012**	**%**	**TLCS**	**TLCS/t**	**TGCS**	**TGCS/t**	**AGCS**	**IF**	**h-index**
1	Social Science & Medicine	32	4.96	169	3.00	293	1.30	494	1.72	1196	125.40	8498	891.24	17.20	2.733	41
2	Health Policy	11	1.71	110	1.95	304	1.35	425	1.48	930	98.63	4169	498.33	9.81	1.550	26
3	Health Affairs	4	0.62	111	1.97	290	1.29	405	1.41	1302	157.44	9034	1163.22	22.31	4.641	45
4	BMC Health Services Research	—	—	—	—	331	1.47	332	1.15	0	0.00	1796	398.64	5.41	1.773	18
5	American Journal of Health-System Pharmacy	—	—	87	1.55	181	0.80	268	0.93	226	31.02	1551	219.67	5.79	1.984	18
6	Medical Care	6	0.93	56	1.00	158	0.70	220	0.76	456	51.17	4744	513.83	21.56	3.227	34
7	Academic Medicine	—	—	80	1.42	131	0.58	211	0.73	219	23.48	2952	330.01	13.99	3.292	29
8	Psychiatric Services	—	—	59	1.05	148	0.66	207	0.72	385	44.77	4012	443.42	19.38	2.013	33
9	BMC Public Health	—	—	—	—	204	0.91	204	0.71	0	0.00	1124	257.93	5.51	2.076	18
10	Health Policy and Planning	—	—	52	0.92	151	0.67	203	0.71	515	103.10	2391	352.32	11.78	3.056	25
11	Total	53	8.22	724	12.86	2191	9.73	2969	10.31	5229	635.01	40271	5068.61	13.56	—	—

TLCS, Total local citation score, which is the number of times cited by other papers in the local collection; TLCS/t, average value of TLCS in a year; TGCS, Total global citation score, which is the citation frequency based on the full WoS count at the time the data was downloaded; TGCS/t, average value of TGCS in a year; AGCS, average citation frequency of an article; IF, impact factor of journals (2012); h-index, h value of journals on HSR.

Major publication outlets of HSR include *Social Science & Medicine, Health Policy,* and *Health Affairs. Health Affairs* ranked first both in quantity and quality with the highest TLCS, TLCS/t, TGCS, TGCS/t, AGCS, impact factor, and H-index, followed by *Social Science & Medicine*. Moreover, *Health Policy* ranked third both in TLCS and TGCS, while *Health Policy and Planning* had a relative high TLCS/t and TGCS/t. In addition, *Medical Care* has a high AGCS and impact factor, which reflects the high quality of articles published in it. Since *Social Science & Medicine, Health Policy, Health Affairs,* and *Medical Care* published health systems-related papers in the early stages, they constitute the most important journals during the development process of HSR (Table 2).

### Countries of publication and collaboration

The publication indicators for the 20 most productive countries/territories in HSR are presented in Table 3. Of these 20 productive countries/territories, 11 were from Europe, 3 from North America, 3 from Asia, 1 from South America, 1 from Oceania, and 1 from Africa. Meanwhile, 16 of these countries are members of the Organization for Economic Co-operation and Development (OECD), while the others are ‘emerging economies’ (such as Brazil, China, and India). Thus, we hypothesize that the economic development and scientific investment has contributed much to the distribution, where all the entire major industrialized countries (G7 countries: USA, UK, Germany, Canada, Italy, France, and Japan) are among the countries within the OECD list. The pattern of publication domination by OECD countries, especially the G7 countries, took place in most scientific fields [44], which reflects the high economic activity and academic level of these countries [45].

**Table 3:** Top 20 most productive countries/territories during 1900-2012

**No.**	**Country**	**1900–1990**	**%**	**1991–2000**	**%**	**2001–2012**	**%**	**1990–2012**	**%**	**TLCS**	**TGCS**	**AGCS**
1	USA	274	42.48	2,536	45.06	8,996	39.96	11,806	41.01	13,047	188,365	15.96
2	UK	22	3.41	368	6.54	2,048	9.10	2,438	8.47	3,046	37,543	15.40
3	Canada	34	5.27	447	7.94	1,946	8.64	2,427	8.43	1,979	29,031	11.96
4	Germany	3	0.47	226	4.02	1,412	6.27	1,641	5.70	624	11,891	7.25
5	Australia	11	1.71	166	2.95	1,244	5.53	1,421	4.94	923	15,040	10.58
6	Brazil	2	0.31	50	0.89	1,266	5.62	1,318	4.58	680	4,833	3.67
7	Spain	3	0.47	104	1.85	805	3.58	912	3.17	349	6,544	7.18
8	Switzerland	16	2.48	62	1.10	629	2.79	707	2.46	1,020	10,904	15.42
9	France	2	0.31	118	2.10	563	2.50	683	2.37	345	6,031	8.83
10	Netherlands	11	1.71	111	1.97	556	2.47	678	2.36	491	8,066	11.90
11	Italy	1	0.16	80	1.42	557	2.47	638	2.22	287	6,842	10.72
12	Sweden	5	0.78	110	1.95	519	2.31	634	2.20	467	8,967	14.14
13	South Africa	11	1.71	51	0.91	416	1.85	468	1.63	707	5,616	12.00
14	China	0	0.00	24	0.43	381	1.69	405	1.41	304	3,339	8.24
15	Denmark	4	0.62	60	1.07	296	1.31	360	1.25	312	5,354	14.87
16	India	3	0.47	21	0.37	315	1.40	339	1.18	295	3,187	9.40
17	Belgium	1	0.16	55	0.98	286	1.27	335	1.16	273	3,764	11.24
18	Mexico	0	0.00	51	0.91	225	1.00	280	0.97	376	2,722	9.72
19	Israel	4	0.62	48	0.85	221	0.98	276	0.96	212	2,255	8.17
20	Norway	1	0.16	39	0.69	235	1.04	275	0.96	216	2,936	10.68
21	Total	408	63.26	4,727	83.99	22,916	101.79	28,041	97.41	25,953	363,230	13.95

TLCS, Total local citation score, which is the number of times cited by other papers in the local collection; TGCS, Total global citation score, which is the citation frequency based on the full WoS count at the time the data was downloaded; AGCS, Average citation frequency of an article.

**Figure 3:**
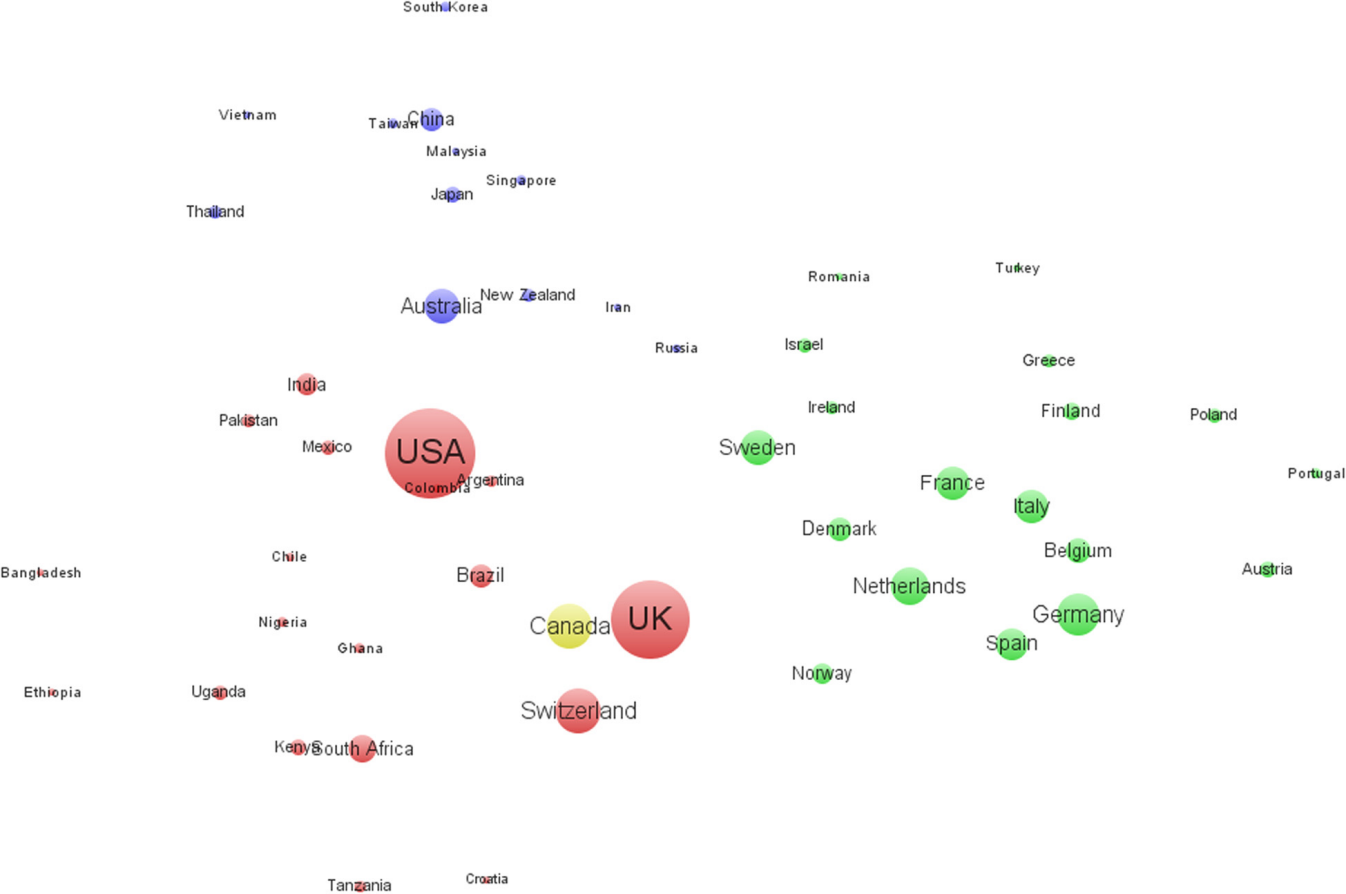
National/territorial collaboration network of the 50 most productive countries.

USA ranked the first in HSR productivity among all countries, with the highest number of articles. The UK published the second highest ratio of articles, followed by Canada, Germany, Australia, and Brazil, while the number of publications for other countries was all below 1,000. Table 3 shows information about the TLCS, TGCS, and AGCS of research articles from the top 20 countries in the global field of HSR. We can observe that USA had the highest TLCS and TGCS, followed by the UK and Canada in turn. The US, Switzerland, and the UK were the front ranking countries in AGCS, showing their superiority in HSR. Denmark and Sweden ranked 15^th^ and 12^th^ in issuing number of articles, respectively, and were the top five in AVGS, which indicates the high average quality of these articles. Brazil ranked 6^th^ in issued volumes with the lowest AGCS, which might indicate considerable problems with the quality of Brazilian issued articles, and the same was the case with Germany and Spain (Table 3).When analyzing the collaboration patterns of the 50 most productive countries/territories with VOSviewer in Figure 3, it was found that some countries/territories with similar properties tend to cooperate in the form of small groups of collaborators, which were clustered into four major of countries/territories: the African and the Americas group (red), European group (green), Asian and Pacific group (purple), and Canada (yellow), each of which usually has several core countries/territories. For example, Australia, China, and Japan are in the core position of the Asian and Pacific group, and Germany and Netherlands are in the core position of the European group. Moreover, apart from the African and the Americas group, the US and UK positions are globally centralized in HSR and can also be observed by their pivotal role in the national collaboration networks as seen in Figure 3. USA and the UK cooperate frequently with other countries/regions and stand in the core position of the entire network, receiving in return mutual benefits from their knowledge transfer among health systems researchers. Other nations, such as Ethiopia, Bangladesh, Portugal, and South Korea, are in the peripheral layer. These countries/territories have less cooperation with other countries/regions, and they are in the outermost layer of the entire cooperation network. As a result, the top two productive countries have carried out most of the international collaborations with other countries in the HSR field (Figure 3).

### Institutes of publication and collaboration

**Figure 4:**
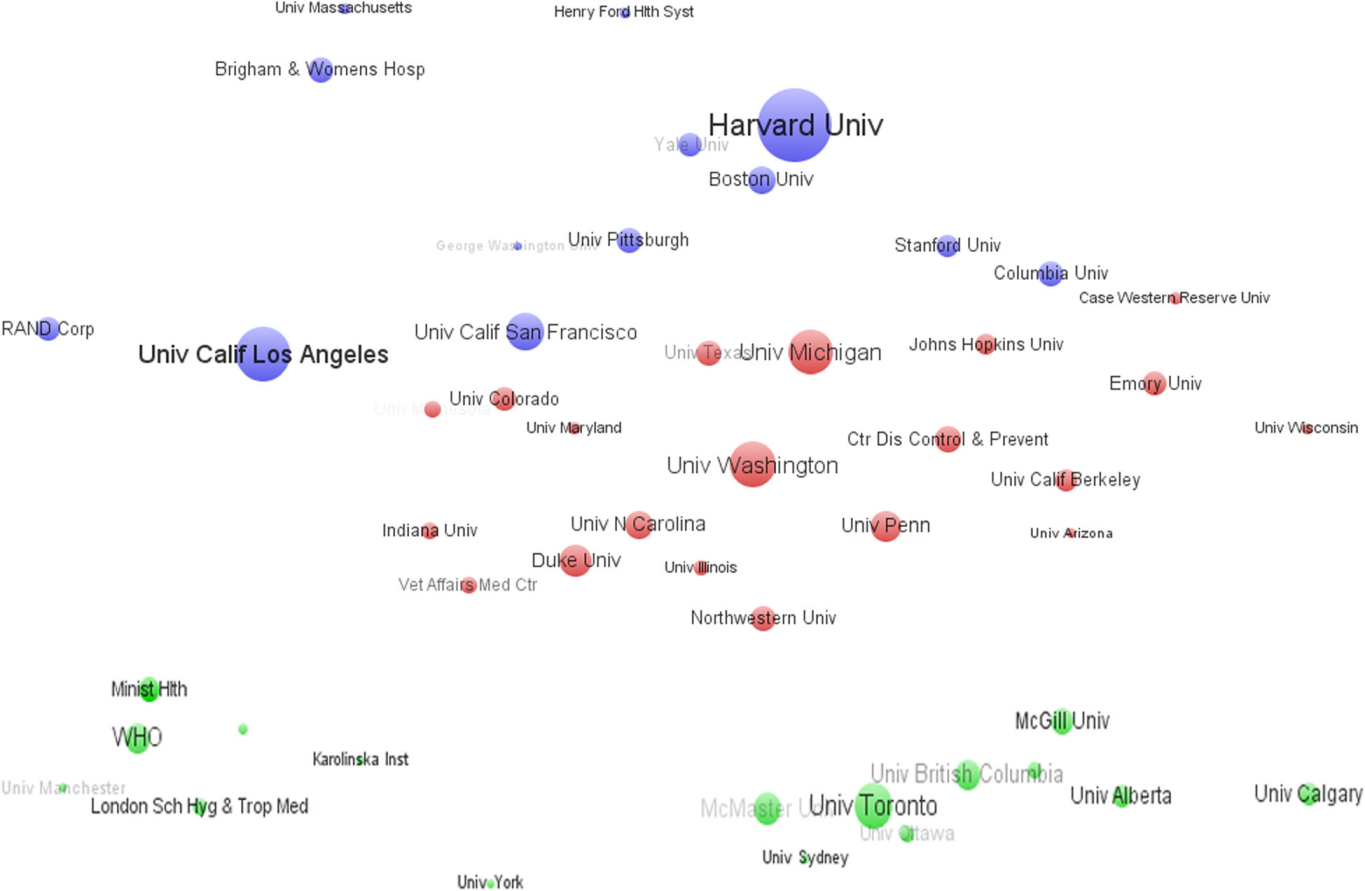
Institutional collaboration network of the 50 most central institutions in health systems research.

The contributions of different institutes are assessed herein by the institutes’ affiliations and with at least one author in the published papers. The top 25 institutes with over 200 papers are ranked by their published articles. From Table 4, we find that Harvard University performed well, and was the most powerful institution in HSR. Harvard University has published 768 articles, i.e., ranking first, followed by University of Toronto, University of Washington, University of Michigan, UCSF, and UCLA. Harvard University was the highest ranking in TLCS and TGCS with more than one thousand citations, followed by WHO, UCSF, and UCLA. Moreover, the WHO has the highest AVGS, followed by UCLA, Center for Disease Control, and UCSF. Harvard University is considered the leading institute in paper quantity, while WHO is the leading institution in article quality. In addition, according to Table 4, 16 institutions are from USA and 3 were Canadian, 1 from the UK, 1 Australian, 1 Brazilian, and 1 Swedish. WHO ranked seventh in the world and also played an important role in HSR reflecting the overall strength of North American and European institutions in this field. International organizations such as the WHO and institutions in the ‘emerging economies’ (such as University of São Paulo) also appear in the productive groups, which reflects that HSR has attracted global attention (Table 4).As in the case with countries/territories, institutions are considered as central participators in institutional collaborations’ networks. When analyzing the collaboration patterns of the 50 most productive institutions with VOSviewer (Figure 4), we found that three major clusters of institutions were generated. The largest cluster was made up of two American institutes’ groups: Harvard University, UCSF, UCLA, University of Michigan, and University of Washington were at the core position of the sub-clusters, respectively. Canadian institutions constituted a similar cluster, and University of Toronto was the core institution of this group. In contrast, the third cluster was made up of institutions form different countries (such as the UK, Australia, Sweden, Brazil) and organizations (such as the WHO). The WHO plays the bridge in increasing the collaboration among these countries and institutions. Thus, the American and Canadian institutions and the WHO are in the core status of the correspondent clusters (Figure 4).

**Table 4:** Top 25 most productive institutions during 1900–2012

**No.**	**Institution**	**1900–1990**	**%**	**1991–2000**	**%**	**2001–2012**	**%**	**1900–2012**	**%**	**TLCS**	**TGCS**	**AGCS**
1	Harvard Univ	17	2.64	120	2.13	631	2.80	768	2.67	1,735	18,732	24.39
2	Univ Toronto	2	0.31	89	1.58	464	2.06	555	1.93	503	7,362	13.26
3	Univ Washington	8	1.24	72	1.28	398	1.77	478	1.66	602	9,677	20.24
4	Univ Michigan	7	1.09	65	1.15	396	1.76	468	1.63	684	9,781	20.90
5	Univ Calif San Francisco	7	1.09	82	1.46	349	1.55	438	1.52	770	11,175	25.51
6	Univ Calif Los Angeles	8	1.24	95	1.69	313	1.39	416	1.45	718	11,168	26.85
7	WHO	3	0.47	43	0.76	285	1.27	331	1.15	1,121	9,141	27.62
8	Univ N Carolina	8	1.24	68	1.21	248	1.10	320	1.11	259	4,303	13.45
9	Johns Hopkins Univ	8	1.24	64	1.14	247	1.10	319	1.11	689	8,105	25.41
10	Columbia Univ	8	1.24	28	0.50	257	1.14	289	1.00	425	5,884	20.36
11	Univ Penn	2	0.31	54	0.96	232	1.03	288	1.00	392	5,069	17.60
12	Ctr Dis Control & Prevent	0	0.00	46	0.82	235	1.04	281	0.98	465	7,431	26.44
13	Stanford Univ	5	0.78	55	0.98	218	0.97	278	0.97	408	6,584	23.68
14	Yale Univ	6	0.93	38	0.68	221	0.98	265	0.92	306	5,009	18.90
15	Boston Univ	6	0.93	30	0.53	222	0.99	258	0.90	375	6,230	24.15
16	Duke Univ	0	0.00	35	0.62	222	0.99	258	0.90	274	4,858	18.83
17	McMaster Univ	3	0.47	53	0.94	205	0.91	255	0.89	260	3,351	13.14
18	Univ Sao Paulo	0	0.00	47	0.84	237	1.05	253	0.88	101	975	3.85
19	Univ British Columbia	4	0.62	47	0.84	216	0.96	248	0.86	181	3,325	13.41
20	Sch Hyg & Trop Med	1	0.16	25	0.44	236	1.05	244	0.85	637	4,571	18.73
21	Minist Hlth	1	0.16	43	0.76	194	0.86	237	0.82	400	2,977	12.56
22	Univ Pittsburgh	1	0.16	35	0.62	197	0.88	233	0.81	242	5,263	22.59
23	Univ Sydney	1	0.16	25	0.44	198	0.88	223	0.77	153	2,633	11.81
24	McGill Univ	2	0.31	28	0.50	178	0.79	208	0.72	127	2,897	13.93
25	Karolinska Inst	1	0.16	25	0.44	179	0.80	204	0.71	165	2,345	11.50
26	Total	116	17.98	1,370	24.34	7,114	31.60	8,516	29.58	12,453	164,858	19.36

TLCS, Total local citation score, which is the number of times cited by other papers in the local collection; TGCS, Total global citation score, which is the citation frequency based on the full WoS count at the time the data was downloaded; AGCS, Average citation frequency of an article.

**Figure 5:**
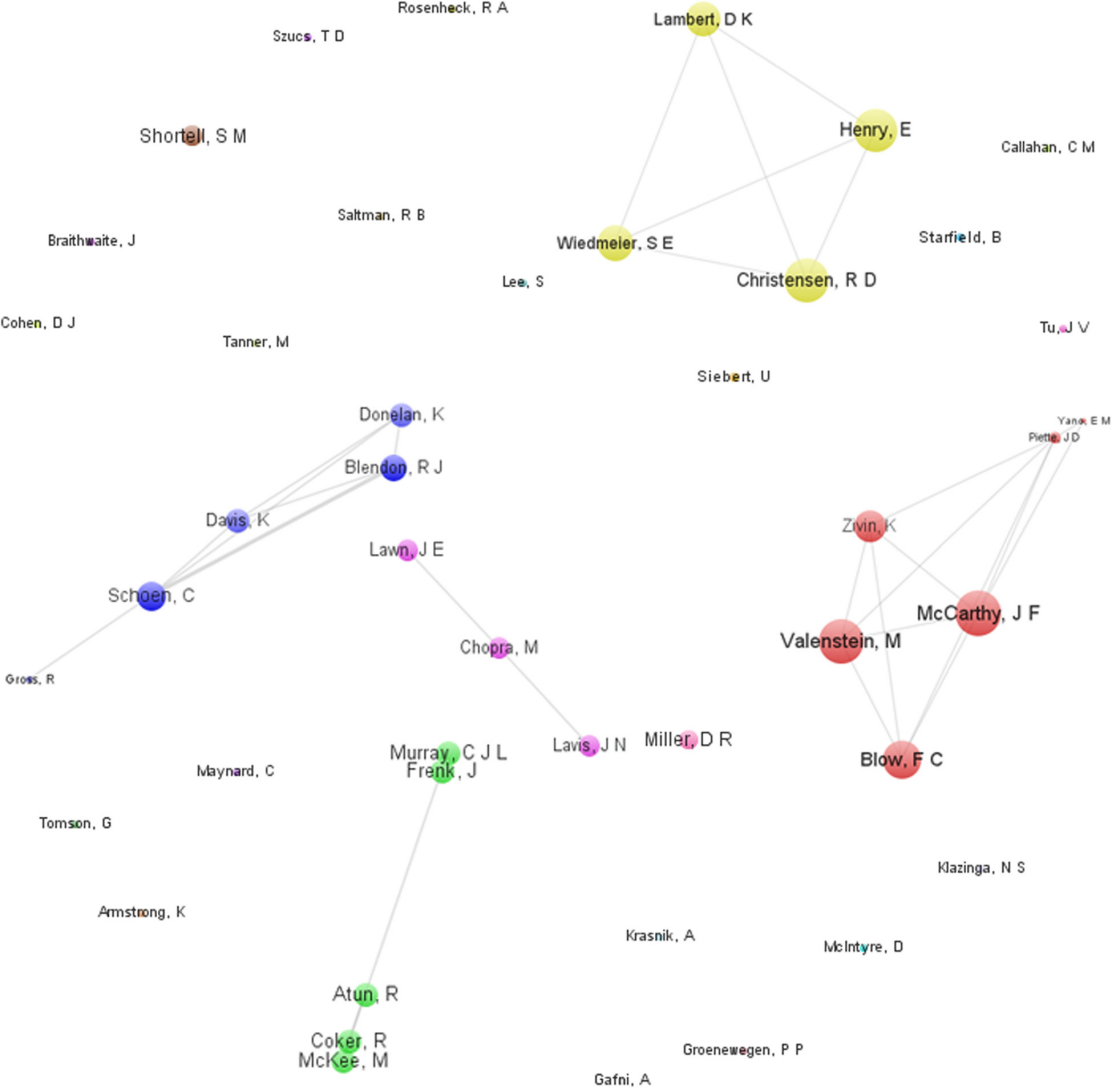
Author collaboration network of the 50 most central institutions in health systems research.

### Authors of publications and collaborations

Consistently with observations in other fields, a small group of productive authors contributed to a significant share of publications in HSR. For example, the top 200 or 0.25% of authors had produced 3,195 or 11.10% of the total health systems articles. The 20 most productive authors identified in the area of HSR and the over 20 published papers are listed in Table 5. These 20 authors together contributed to the publication of 628 papers, i.e., an average of 31.40 papers per author, which accounted for a 2.18% share in the cumulative worldwide publication output during the period 1900–2012. The most productive authors in HSR were McKee M with 48 articles, followed by Valenstein M, McCarthy JF, Christensen RD, Henry E, and Tanner M. Considering the quality/impact of papers, these 20 most productive authors have received a total of 13,918 citations for the 628 papers they have published with an average of 22.16 citations per article. Murray CJL’s 26 works had 1,407 global citations score, followed by Piette JD, Miller DR, Davis K, Starfield B, and Frenk J. With regards to the local citations score, Murray CJL had the highest local citations score of 248, followed by Frenk J, McKee M, and Piette JD. Moreover, in the AGCS, which is sorted in descending order, Murray CJL ranked first, followed by Piette JD, Miller DR, Starfield B, Davis K, and Chopra M. Considering the fact that older articles are likely to have more citations, we also calculated the TLCS/t and TGCS/t of every author (Table 5). There was a high proportion of American researchers (17 out of 23) in the top 20 list, which suggests that there are many active researchers conducting health systems studies in the USA, a country with high health expenditures (Table 5).

**Table 5:** Top 20 most productive authors during 1900–2012

**No.**	**Author**	**Institute**	**Recs**	**%**	**TLCS**	**TLCS/t**	**TGCS**	**TGCS/t**	**AGCS**
1	McKee M	Univ London London Sch Hyg & Trop Med	48	0.17	162	23.40	690	116.13	14.38
2	Valenstein M	Univ Michigan; SMITREC, Ann Arbor Ctr Clin Management Res, Dept Vet Affairs	38	0.13	94	20.11	554	111.20	14.58
3	McCarthy JF	Univ Michigan; SMITREC, Ann Arbor Ctr Clin Management Res, Dept Vet Affairs	36	0.13	97	20.76	619	118.99	17.19
4	Christensen RD	Department of Women and Newborns, Intermountain Healthcare; Ogden McKay-Dee Hospital Center	33	0.11	79	19.94	429	98.46	13.00
5	Henry E	Institute for Health Care Delivery Research, Intermountain Healthcare	31	0.11	78	19.19	431	100.71	13.90
6	Tanner M	Univ BaselSwiss, Tropical and Public Health Institute	31	0.11	32	4.12	564	99.33	18.19
7	Piette JD	Univ Michigan; Vet Affairs Ann Arbor Ctr Clin Management Res & D, Ctr Excellence	29	0.10	158	18.23	1,353	157.71	46.66
8	Rosenheck RA	Yale Univ; VA New England Mental Illness Res Educ & Clin Ctr	29	0.10	81	8.52	615	66.21	21.21
9	Blow FC	Univ Michigan; SMITREC, Ann Arbor Ctr Clin Management Res, Dept Vet Affairs	28	0.10	76	16.91	557	99.20	19.89
10	Frenk J	Harvard Univ, Sch Publ Hlth	27	0.09	245	25.97	710	92.37	26.30
11	Braithwaite J	Univ New S Wales, Fac Med, Australian Inst Hlth Innovat, Ctr Clin Governance Res	26	0.09	23	5.62	174	45.03	6.69
12	Davis K	Commonwealth Fund, Res & Evaluat	26	0.09	135	16.72	833	95.37	32.04
13	Murray CJL	Univ Washington, Inst Hlth Metr & Evaluat, Dept Global Hlth	26	0.09	248	39.51	1,407	306.76	54.12
14	Miller DR	Boston Univ, Sch Publ Hlth; Edith Nourse Rogers Mem Vet Adm Hosp, Ctr Hlth Qual Outcomes & Econ Res	24	0.08	129	12.58	1,058	120.73	44.08
15	Alexander JA	Univ Michigan, Sch Publ Hlth, Dept Hlth Management & Policy	23	0.08	34	3.48	191	25.61	8.30
16	Shortell SM	Univ Calif Berkeley, Sch Publ Hlth	23	0.08	104	7.65	596	51.72	25.91
17	Klazinga NS	Univ Amsterdam, Acad Med Ctr, Dept Publ Hlth; Org Econ Co operat & Dev	22	0.08	29	3.67	237	32.63	10.77
18	McIntyre D	Univ Cape Town, Fac Hlth Sci, Dept Publ Hlth & Family Med, Hlth Econ Unit	22	0.08	78	21.08	263	62.61	11.95
19	Starfield B	Johns Hopkins Bloomberg Sch Publ Hlth, Dept Hlth Policy & Management	22	0.08	147	15.10	720	75.52	32.73
20	Armstrong K	Univ Penn, Abramson Canc Ctr	21	0.07	76	10.84	448	59.01	21.33
20	Chopra M	Univ Western Cape; MRC, Hlth Syst Res Unit	21	0.07	134	26.40	656	124.79	31.24
20	Lee A	Chinese Univ Hong Kong, Sch Publ Hlth & Primary Care, Sch Publ Hlth, Prince Wales Hosp	21	0.07	81	6.42	602	51.14	28.67
20	Zivin K	SMITREC, Ann Arbor Ctr Clin Management Res, Dept Vet Affairs	21	0.07	53	12.45	211	52.32	10.05
21	Total		628	2.18	2,373	358.67	13,918	2,163.55	22.16

Recs, Number of articles; %, Percentage of articles; TLCS, Total local citation score, which is the number of times cited by other papers in the local collection; TLCS/t, Average value of TLCS in a year; TGCS, Total global citation score, which is the citation frequency based on the full WoS count at the time the data was downloaded; TGCS/t, Average value of TGCS in a year.

We also analyzed the collaboration patterns of the 50 most productive authors with VOSviewer, and the collaboration map is presented in Figure 5. We noticed that several authors tended to cooperate with a small group of collaborators, generating five major clusters of authors, each usually had one or two core authors. Others were singles in the productive authorship collaboration network. According to the social network analysis, it proved that the research collaboration in HSR is not tight. The component analysis found that five research groups can be regarded as the backbone in this field. Therefore, researchers in HSR should strengthen their collaboration to improve the development and academic level of this field (Figure 5).

### Citation of research papers

The total citation count obtained from the WoS database shows that the total time that a particular article was cited by other research work is listed in this database. The number of citations does not necessarily indicate the quality of a paper, but it is a measure of its impact and/or visibility in this field. The top 11 most frequent cited articles that were selected (LCS ≥48 times) from 1900 to 2012 are listed in Table 6. The most frequent cited article was ‘*The De Facto US Mental and Addictive Disorders Service System*’. ‘*Epidemiologic Catchment Area Prospective 1-Year Prevalence Rates of Disorders and Services*’ written in 1993 by Regier DA from the National Institutes of Health of USA, has been cited 1,183 times since being published in the journal *Archives of General Psychiatry*, which vastly exceeds the citation of other articles. Meanwhile, Evans T from the WHO had the highest contribution (No. 10 and 11) of articles among the 11 most frequently cited articles, which also exhibited its predominance. In addition, among the top 11 cited papers, USA contributed to 7 and Switzerland 3 articles, respectively, and Brazil, a developing country and an ‘emerging economy’, held 1. The WHO published 3 articles and ranked first among all institutions, which reflects its dominant position in the HSR (Table 6).

**Table 6:** Top 11 most cited health systems research papers

**No.**	**Title**	**Authors**	**Journal**	**Year**	**Times cited (LCS/GCS/GCS/t)**	**Institute (first)**	**Country**
1	Satisfaction with health systems in ten nations	Blendon RJ, Leitman R, Morrison I, Donelan K	Health Affairs	1990	48/132/5.74	Harvard University	USA
2	The de facto US mental and addictive disorders service system. Epidemiologic catchment area prospective 1-year prevalence rates of disorders and services	Regier DA, Narrow WE, Rae DS, Manderscheid RW, Locke BZ, Goodwin FK	Archives of general psychiatry	1993	57/1183/59.15	National Institutes of Health	USA
3	Correction: The deteriorating administrative efficiency of the United States health care system	Woolhandler S, Himmelstein DU	The New England journal of medicine	1994	50/242/11.00	National Library of Medicine	USA
4	A framework for assessing the performance of health systems	Murray CJ, Frenk J	Bulletin of the World Health Organization	2000	59/129/9.92	World Health Organization	Switzerland
5	Race and trust in the health care system	Boulware LE, Cooper LA, Ratner LE, LaVeist TA, Powe NR	Public Health Reports	2003	51/225/22.50	Johns Hopkins University	USA
6	Effect of the transformation of the Veterans Affairs Health Care System on the quality of care	Jha AK, Perlin JB, Kizer KW, Dudley RA	The New England journal of medicine	2003	84/332/33.20	Veterans Health Administration	USA
7	The contribution of primary care systems to health outcomes within Organization for Economic Cooperation and Development (OECD) countries, 1970–1998	Macinko J, Starfield B, Shi L	Health services research	2003	49/168/16.80	National School of Public Health	Brazil
8	Household catastrophic health expenditure: a multi-country analysis	Xu K, Evans DB, Kawabata K, Zeramdini R, Klavus J, Murray CJ	Lancet	2003	51/281/28.10	World Health Organization	Switzerland
9	Comparison of quality of care for patients in the Veterans Health Administration and patients in a national sample	Asch SM, McGlynn EA, Hogan MM, Hayward RA, Shekelle P, Rubenstein L, Keesey J, Adams J, Kerr EA	Annals of internal medicine	2004	48/247/27.44	University of California, Los Angeles	USA
10	Human resources for health: overcoming the crisis	Chen L, Evans T, Anand S, Boufford JI, Brown H, Chowdhury M, Cueto M, Dare L, Dussault G, Elzinga G, Fee E, Habte D, Hanvoravongchai P, Jacobs M, Kurowski C, Michael S, Pablos-Mendez A, Sewankambo N, Solimano G, Stilwell B, de Waal A, Wibulpolprasert S	Lancet	2004	66/289/32.11	Harvard University	USA
11	Overcoming health-systems constraints to achieve the Millennium Development Goals	Travis P, Bennett S, Haines A, Pang T, Bhutta Z, Hyder AA, Pielemeier NR, Mills A, Evans T	Lancet	2004	72/221/24.56	World Health Organization	Switzerland

### Keywords and co-words analysis

To locate the most popular research topics and their trends, the distribution of authors’ keywords and keywords-plus was investigated. As for author keywords analysis, they offer information about research trends from the view of researchers, and they have proved to be important in monitoring the development of science [46]. Keywords-plus supplies additional search terms extracted from articles’ titles cited by authors in their bibliographies and footnotes [47]. Therefore, the topic of papers can be obtained from the authors’ keywords and keywords-plus by cluster analysis. Hence, we performed keyword analysis to gain insights about HSR trends and frontiers.

### Distribution of author keywords

**Figure 6:**
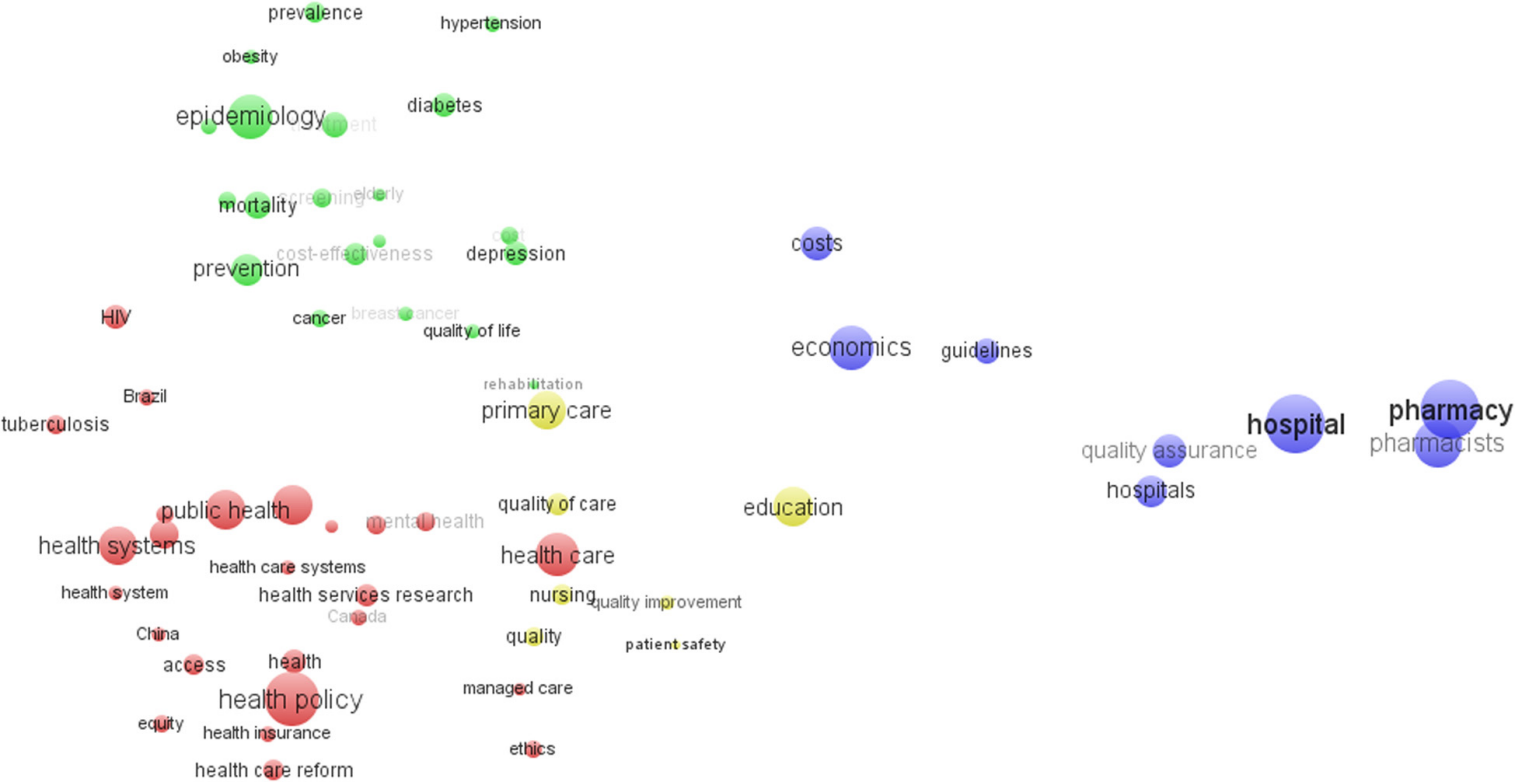
Co-words cluster map of author keywords.

Examination of author keywords in this study period revealed that, altogether, 29,480 author keywords were used, among which 21,658 (73.47%) keywords appeared only once, and 3,188 (10.81%) keywords appeared twice. The high percentage of only once author’s keywords probably indicated to the lack of continuity in research and a wide disparity in research focus. Another reason was that these keywords might not be standard or widely accepted by researchers. Author keywords appearing in articles that refer to HSR were calculated, and the top 60 author keywords were used and clustered with VOSviewer from 1900 to 2012 (Figure 6). The top three most frequently used keywords were ‘health policy’, ‘epidemiology’, and ‘health care’, which was highly accorded with the research trend as we know. The 60 author keywords were divided into four groups, and represent the hot research areas of HSR.

Group 1 (red) includes health policy and analysis research such as policy design and implementation [48-50], challenges of health policy and health technology [51,52], health care system reforms and performance monitoring [53-56] (i.e., China), metropolitan and regional health planning [57], priority setting and agendas [58,59], policy of financial access and equity [48]. New health policies represent the efforts in introducing deliberate and purposeful changes within health systems. Ideas and concepts’ analysis are related to such policy and are important parts of HSR. When seeking support for better policy implementation, it is critical that we understand the factors that influence the policy outcomes. Through understanding the nature of the policy and the processes of policy changes we could gain new insights that help to explain how health systems actors and the relationships of power and trust among them influence health systems performance. Health systems and sub-systems research includes health systems frameworks [60,61], health systems management [62,63], health systems strengthening [13,17,21], health systems evaluation [48,64], accessibility, equality and efficiency of health systems [65-67], primary care system [68], public health systems [69-71], and mental health systems [72-74]. Healthcare and services research (i.e., Brazil) include accessibility, equality and efficiency of healthcare [75,76], primary healthcare and mental healthcare [49,73,74,77-79], managed care and integrated care [80-82], healthcare innovation [83,84], health care delivery models [85,86], responsiveness to health services [53,87], influencing demand for care [88,89], financial question of healthcare [48,90]. Health includes health measurement and evaluation [91,92], health promotion [93], health accessibility and equality [94-96].

Group 2 (green) includes epidemiology and economics of communicable diseases, such as HIV, tuberculosis, and malaria, and non-communicable diseases, such as hypertension, diabetes, cancer, obesity, global diseases [4,21,97], especially in elderly and children, and developing countries.

Group 3 (yellow) includes primary care research, training and education [98-100], quality of care [101,102], quality improvement [103], patient safety improvement and management [104], primary care expenditure [105], general practitioner [106], contribution of primary care to health systems and health [68], primary care reform and evaluation, family medicine [107,108], health literacy [67,109,110], chronic disease management [111], and integrated care.

Group 4 (blue) includes health economics and health costs, health expenditure control [112,113], health economic evaluation [114,115], costs of disease and care, economic burden of disease [4,58]. Pharmacy of hospital includes pharmacy practice in hospital [116,117], pharmacy residency in hospital [118,119], pharmacy practice model [120,121], monitor of health-system pharmacy [122,123], and access, quality, and safety of medicines [124]. These four groups contain eight topics, which are the major hotspots in all HSR topics and are thus considered to be the basic academic trends as shown in Figure 6.

### Distribution of keywords-plus

**Figure 7:**
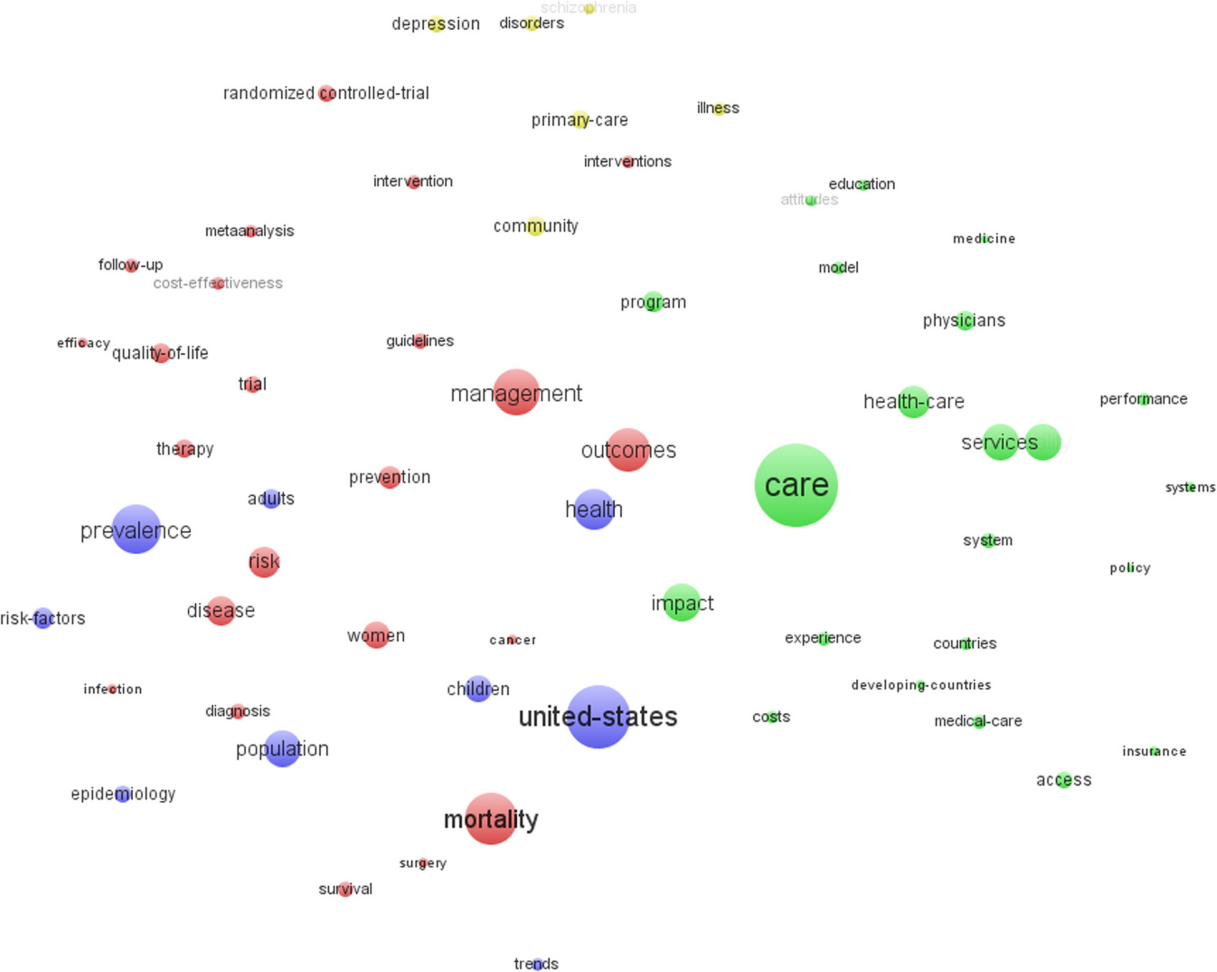
Co-words cluster map of keywords plus.

As supplies of author keywords, we also examined the co-occurrence relationships among the top 60 high frequency keywords-plus and the co-word networks were visualized using VOSviewer (Figure 7). With the analysis of keywords-plus, again, the top three most frequently used keywords-plus were ‘care’, ‘United States’, and ‘mortality’. The similarities between author keywords and keywords-plus could be listed. Similarly to the map of author keywords, ‘policy’, ‘system’, ‘health’, ‘care’, ‘health care’, ‘services’, ‘epidemiology’, ‘prevention’, ‘risk factors’, ‘mortality’, ‘children’, ‘adults’, ‘insurance’, ‘primary care’, ‘education’, ‘quality of life’, ‘access’, ‘cost-effectiveness’, ‘costs’, ‘developing-countries’, and ‘medicine’ also appeared in the top 60 most frequently used keywords (Figure 6). ‘Meta-analysis’ and ‘randomized controlled trial’ were not in the map of top author keywords, but also had significant roles in the map. That means these analytical methods were frequently used in HSR. Moreover, ‘disorders’ and ‘schizophrenia’ show that mental health research was also a hot topic in recent years. In addition, research interests that are related to the USA received relatively more attention. Finally, there were clearly increasing research interests in ‘efficacy’ as well as in topics related to ‘community’, ‘physicians’, and ‘women’ judging by the relatively higher ranking of these keywords. They represented the research emphasis of HSR (Figure 7).

## Conclusions

In this study, we have provided a supplemental evaluation of the status of HSR. Our analysis confirms that papers in HSR have increased rapidly during the last 20 years, and most notably in the last 8 years. In total, there were 28,787 research articles published in 3,674 journals listed in 140 SCI subject categories. Research in the fields of HSR have mainly focused on public, environmental and occupational health, health care sciences and services, and general and internal medicine. All output has been concentrated in several journals such as *Social Science and Medicine, Health Policy, Health Affairs,* and *BMC Health Services Research*. Hence, these journals are the core journals and play important roles during the knowledge dissemination and exchange in HSR.

The HSR output is distributed unevenly by countries, institutes, and authors. OECD countries, especially the G7 countries, have published the majority of articles. In addition, USA, UK, and Canada stand in the core of international collaborative networks. Thus, they promote the creation, transmission, and sharing of knowledge in HSR fields. China, a developing country, also plays an important role in the country’s collaborative network category. Furthermore, American and Canadian institutions, and the WHO have made great advances in paper research production, citation, and cooperation, along with the overall great strengths and good development prospects. Meanwhile, the most frequently cited articles come from the USA and Switzerland, during which American-authored papers had contributed the most to this field. Brazil, a developing country and as an emerging economy, also held one. The WHO published three articles and ranked first among all institutions, reflecting its dominant position in the HSR. McKee M, from the London School of Hygiene & Tropical Medicine published the most articles. However, most of the productive authors are from American institutes, such as University of Michigan. Finally, it could be concluded that the USA and its universities and academic institutions play a dominant role in the production, collaboration, citation, and high quality of articles.

HSR is an interdisciplinary area and includes medicine, public-environmental and occupational health, health care sciences and services, pharmacology and pharmacy, economics, sociology, information science and technology, and psychology. Whilst health economics is a central discipline of HSR, the analyses most centrally fell within HSR, including work that focuses on financing. Its current hotspots center on health policy and analysis research, health systems and sub-systems research, healthcare and services research, epidemiology/economics of communicable and non-communicable global diseases, primary care research, health economics and health costs, pharmacy of hospital, and health (such as health measurement and evaluation, health promotion, health accessibility and equality). Meanwhile, the main topics found from the analysis of keywords-plus are in accordance with authors’ keywords analysis results. Further, ‘meta-analysis’, ‘randomized controlled-trial’, ‘disorders’, ‘schizophrenia’, ‘United States’ ‘physicians’, and ‘women’, which were not in the top author keywords map, but also had significant roles in the keywords-plus map. Hence, these topics are also hot topics in HSR. Furthermore, from the perspective of citation, most researchers that have studied health systems have concentrated on the topics of health outcomes (i.e., health outcomes measurement), responsiveness to health systems (i.e., trust and satisfaction), leadership and governance (i.e., priority setting, performance monitoring and accountability arrangements), health financing and health expenditure (i.e., catastrophic health expenditure, and protected from financial catastrophe), health services and quality of care (i.e., access, quality, safety, and continuity), health performance assessment (i.e., framework), health information systems (i.e., electronic medical record), and health workforce. In addition, more and more attention has been paid to the developing countries, especially the ‘emerging economies’ (such as Brazil and China).

These findings will provide evidence of the current status and trends in HSR all over the world, as well as clues to the impact of this popular topic, thus helping scientific researchers understand the panorama of HSR, and predict the dynamic directions of research. Therefore, based on these findings, policy makers could understand the status and positions of their countries or institutions, and the directions of HSR all over the world. Thus, they could spell out suggestions for HSR or reform directions. For example, they could develop post-MDG global health agendas, set health systems priorities areas, strengthen fragile blocks of health systems, and learn successful lessons from abroad to achieve universal health coverage. Meanwhile, the process of developing an HSR study begins with identifying the topic of focus – the issue or problem you want to investigate – and the related questions. Hence, with the help of these findings, researchers could select their research directions or topics, cooperative institutions and partners, and even choose academic achievements’ platform exchange. In addition, because HSR is defined by the topics and the questions it addresses rather than the disciplinary perspective or the particular approach to data collection and analysis it adopts, the distribution of research subjects and hot topics will help people to understand the concepts of health systems.

The results presented herein can provide evidence about the current status and future trends in HSR, as well as clues to the impact of this hot topic. However, they could not present the research foci of HSR simultaneously from multiple angles. For example, we could not clearly present the research features of journals, countries, institutions, or authors in one knowledge map. Further, we could not show the evolution pathway of HSR from different angles, such as the topic changes with time. Thus, future efforts are needed to describe the features of journals, countries, institutes, and authors, specifically the performance changes of processes in health systems areas.

## Abbreviations

AGCS: average global citation score; AHPSR: Alliance for Health Policy and Systems Research; HSR: Health Systems Research; MDGs: Millennium Development Goals; OECD: Organization for Economic Co-operation and Development; TGCS: total global citation score; TLCS: total local citation score; WHO: World Health Organization’s; WoS: Web of Science.

## Competing interests

The authors declare that they have no competing interests.

## Authors’ contributions

LY initiated and designed the study, she also obtained the funding. QY, PHL and ZYL were involved in the data collection and analysis. QY participated in the study design, collected the data, and conducted the data analysis and the writing of the manuscript. PHL contributed to the design and analysis of the data and prepared the manuscript. ZYL contributed to the data analysis and revision of the manuscript. KC, FL, SQC, LYH and TAY edited the paper. All authors were involved in the interpretation of data and have read and given final approval of this paper.

## Supplementary Material

Additional file 1 Annex S1. Click here for file
