# Supplementary material for: Scientometric trends and knowledge maps of global health systems research
Source: Health Res Policy Syst. 2014 Jun 5;12:26. doi: 10.1186/1478-4505-12-26 (PMC4065315; doi:10.1186/1478-4505-12-26)
Supplement: Additional file 1 — Annex S1. [file 1478-4505-12-26-S1.docx]

| Health system building blocks and their sub-components |
| --- |
| **Service delivery** |
| – Access, integrated care, continuum of care and modes of delivery |
| – Non-state sector (e.g., contracting, private sector) |
| – Quality of care and performance |
| **Human resources Medicines** |
| – Distribution and retention |
| – Training (pre-service and in-service) |
| – Migration |
| **Information systems** |
| – Medical and drug records; Computerized records; and management information systems |
| **Medicines** |
| – Monitoring (e.g., adverse reactions) |
| – Selection (e.g., in essential drug lists) |
| – Regulation and Quality Assurance |
| – Intellectual Property |
| – Access |
| – Policy/Reform (e.g., national drug policies) |
| – Insurance and Financing |
| – Medicine Supply (e.g., forecasting) |
| – Prescribing and Utilization |
| – Information (e.g., for education and advocacy) |
| – Marketing(e.g., drug promotion) |
| **Health financing** |
| – Payment mechanisms |
| – Health insurance |
| – Resource allocation |
| **Governance and leadership** |
| – Government regulation and legislation |
| – Licensing and accreditation |
| – Professional authority and roles (e.g., scope, content and location of practice) |
| – Audit Service delivery |
| – Consumer involvement |

# Service Delivery

## Access, integrated care, continuity of care, modes of delivery

### TS="Delivery of Healthcare" OR "Health Care Deliver*" OR "Healthcare Deliver*" OR "Health Care" OR "Community Based Distribution*" OR "Community-Based Distribution*" OR "Contraceptive Distribution*" OR "Delivery of Dental Care" OR "Dental Care Delivery" OR "Distributional Activit*" OR "Nonclinical Distribution*" OR "Non-Clinical Distribution*" OR "Integrated Delivery System*" OR "Integrated Health Care System*" OR "Consultation and Referral" OR "Health Service* Gatekeeper*" OR "Referral*" OR "Second Opinion*" OR "Consultation*" OR "Hospital Referral*" OR "Out-of-Hours Medical Care" OR "Out of Hours Medical Care" OR "After Hours Care" OR "Health Care Reform*" OR "Healthcare Reform*" OR "Health Service* Need*" OR "Health Service* Demand*" OR "Target Population*" OR "Availability of Health Service*" OR "Health Service* Availability" OR "Accessibility, Health Service*" OR "Access* to Health Care" OR "Accessibility of Health Service*" OR "Health Service* Geographic Accessibility" OR "Program Accessibility" OR "Contraceptive Availability"

## Role of non-state sector

### TS="Private Practice*" OR "Solo Practice*" OR "Family Physician*" OR "General Practitioner*" OR "General Practice* Physician*" OR "Professional Corporation*" OR "Corporate Practice*" OR "Non-Profit Organization*" OR "Non Profit Organization*" OR "Nonprofit Organization*" OR "Outsourced Service*" OR "Outsourcing" OR "Social marketing"

### TS="private sector" OR "private practitioner" OR Private practice OR "private provider" OR "private providers" OR "private provision" OR "Non state sector" OR "Non state" OR "non formal" OR informal OR traditional OR licensed OR "non licensed" OR unlicensed OR "drug vendors" OR "medicine sellers" OR pharmacists OR "NGO" OR "Public Private Partnership" OR Franchising OR Franchizing OR Contract* OR Contracts OR Contracting

### #2 NOT#3

## Quality of care and performance

### TS="health manpower" OR "Health Occupations Manpower" OR "Health Workforce"

### TS="health personnel" OR "health care personnel" OR "healthcare personnel" OR "medical personnel" OR "health professional" OR "health care professional*" OR "healthcare professional*" OR "medical professional*" OR "health worker*" OR "health care worker*" OR "healthcare worker*" OR "medical worker*" OR "health workforce" OR "health care workforce" OR "healthcare workforce" OR "medical workforce" or “human resource”

### #6 NOT #5

### #5 OR #7

### TS="Healthcare Quality Assurance*" OR "Health Care Quality Assurance*" OR "Healthcare Quality Assessment*" OR "Health Care Quality Assessment*" OR "Healthcare Quality Indicator*" OR "Health care Quality Indicator*" OR "Quality Indicator*" OR "Health Care Quality" OR "Quality of Healthcare" OR "Healthcare Quality" OR "Quality Control*" OR "Total Quality Management" OR "Continuous Quality Management" OR "Health Care Evaluation Mechanism*"

### #8 AND #9

Service Delivery (All Categories)

### (#1 NOT #4) AND (#1 NOT #10)

### (#4 NOT #1) AND (#4 NOT #10)

### (#10 NOT #1) AND (#10 NOT #4)

### (#11) OR (#12) OR (#13)

# Health Workforce

### TS="health manpower" OR "Health Occupations Manpower" OR "Health Workforce"

### TS="health personnel" OR "health care personnel" OR "healthcare personnel" OR "medical personnel" OR "health professional" OR "health care professional*" OR "healthcare professional*" OR "medical professional*" OR "health worker*" OR "health care worker*" OR "healthcare worker*" OR "medical worker*" OR "health workforce" OR "health care workforce" OR "healthcare workforce" OR "medical workforce" or “human resource”

### (#2) NOT (#1)

### (#1) OR (#3)

## Distribution

### TS="Health Services Need*" OR "Target Population" OR "Health Care Rationing" OR "Healthcare Rationing" OR "Resource* Allocation*" OR "Allocation of Resource*" OR "Personnel Management" OR "Client-Staff Ratio*" OR "Client Staff Ratio*" OR "Hospita*l Personnel Management" OR "Hospital* Personnel Administration*" OR "Hospital* Personnel Organization*" OR "Health Resource*" OR "Job Satisfaction*" OR "Work Satisfaction*" OR "Professional Burnout" OR "Personnel Turnover*" OR "Employee Turnover*" OR "Personnel Staffing" OR "Personnel Scheduling" OR "Staffing and Scheduling" OR "Scheduling and Staffing" OR "Career Mobilit*" OR "Clinical Ladder*" OR "Job Ladder*" OR "Career Ladder*" OR "Personnel Selection*" OR "Recruitment Activit*" OR "Personnel Recruitment*"

### TS=supply OR shortage OR capacity OR employment OR distribution OR maldistribution OR maldistributions OR recruit OR recruite OR recruitement OR recruiting OR allocation OR reallocation OR mobility OR practice OR rural OR remote OR underserved OR (imbalance OR retention OR "retention strategy" OR "financial incentive" OR "monetary incentive" OR allowances OR benefits OR "compulsory service" OR "bonding scheme" OR "vacancy rates" OR motivation

### #6 NOT #5

### #5 OR #7

### #4 AND #8

## Migration

### TS= "Emigration and Immigration" OR "Immigration and Emigration" OR "Chain Migration*" OR "Chain Migrations" OR "Emigration*" OR "Immigration*" OR "In-Migration*" OR "In Migration*" OR "Turnaround Migration*" OR "Labor Migration*" OR "Out-Migration*" OR "Out Migration*" OR "Return Migration*" OR "Settlement and Resettlement" OR "Resettlement and Settlement" OR "Temporary Migration*" OR "Border Crossing*" OR "International Migration*" OR "Foreign Professional Personnel " OR "International Educational Exchange*" OR "Foreign Medical Graduate*"

### TS=emigration OR immigration OR migration OR "brain drain" OR "border crossing"

### #11 NOT #10

### #10 OR #12

## Education I (undergraduate, graduate training)

### TS= "Student*" OR "School Enrollment*" OR "Public Health Student*" OR "Health Occupations Student*" OR "Premedical Student*" OR "School Dropout*" OR "Health Personnel" OR "Health Care Provider*" OR "Healthcare Provider*" OR "Fieldworker*" OR "Field Worker*"

### TS="female doctor" OR "male nurse"

### #15 NOT #14

### #14 OR #16

### TS="Scholarship* and Fellowship*" OR "Scholarship*" OR "Fellowship*" OR "Training Support*" OR "Educational Subsid*" OR "Traineeship*" OR "Educational Grant*" OR "Student Loan*" OR "Health Planning Support*" OR "Health Planning Subsid*" OR "Public Financing" OR "Government Financing" OR "Government Subsid*" OR "Hill-Burton Act*" OR "Hill Burton Act*" OR "Federal Aid*" OR "Organized Financing" OR "Grant*" OR "Community Financing" OR "Financial Support*" OR "Personnel Selection*" OR "Recruitment Activit*" OR "Personnel Recruitment*" OR "Education*" OR "Workshop*" OR "Training Program*" OR "Educational Activit*" OR "Literacy Program*" OR "Distance Education" OR "Distance Learning" OR "Correspondence Course*" OR "Nursing Diploma Program*" OR "Curriculum*" OR "Curricula" OR "Short-Term Course*" OR "Short Term Course*"

### #17 AND #18

## Education II-(pre-service)

### TS="pre service" OR preservice OR "Inservice Training" OR "On-the-Job Training" OR "On the Job Training" OR " Employee Orientation Program*"OR "continuing education"

### #4 AND #20

Human Resources for Health (All Categories)

### (#9 NOT #13) AND (#9 NOT #19) AND (#9 NOT #21)

### (#13 NOT #9) AND (#13 NOT #19) AND (#13 NOT #21)

### (#19 NOT #9) AND (#19 NOT #13) AND (#19 NOT #21)

### (#21 NOT #9) AND (#21 NOT #13) AND (#21 NOT #19)

### #22 OR #23 OR #24 OR #25

# Health Information systems

### TS="Health Record*" OR "Personal Health Record*" OR "Personal Medical Record*" OR "Personal Electronic Health Record*" OR "Computerized Patient Record*" OR "Electronic Medical Record*" OR "Electronic Health Record*" OR "Medical Record*" OR "Medical Transcription*" OR "Health Diaries" OR "Health Diary" OR "Automated Medical Record*" OR "Automated Medical Record* System*" OR "Computerized Patient Medical Record*" OR "Computerized Medical Record*" OR "Computerized Medical Record* System*" OR "Problem-Oriented Medical Record*" OR "Problem Oriented Medical Record*" OR "Management Information System*" OR "Drug Information Service*"

# Medicines

### TI=(drug or drugs or pharmaceutical or pharmaceuticals or medicines or medicine)

### TI=(drug or drugs or pharmaceutical or pharmaceuticals or medicines or medicine)

### TI=pharmaceutical or pharmaceuticals or medicines)

### TS= (pharmaceutical or pharmaceuticals or medicines)

## Regulation

### TS="Narcotic and Drug Control*" OR "Pharmaceutical Polic*" OR "Pharmaceutic Polic*" OR "Narcotic Control*" OR "Drug Control*" OR "Drug Regulation*" OR "Drug Product Labeling" OR "Drug Package Insert*" OR "Drug Labelling") AND ("Court decision*" OR "Government regulation*" OR "Judicial aspect*" OR "Juridical aspect*" OR "Statute*" OR "Lawsuit*" OR "Legal aspect*" OR "Jurisprudence" OR "Litigation" OR "Medicolegal aspect*" OR "Ordinance*" OR "Regulation*" OR "Civil suit*" OR "Law*" OR "Situational Ethic*" OR "Moral Polic*" OR "Natural Law*" OR "Egoism" OR "Metaethic*" OR "Ethical Issue*"

## Monitoring

### TS="Drug Monitoring" OR "Adverse Drug Reaction Reporting"

### TS=Pharmacovigilance

### (#6) NOT (#7)

### (#7) OR (#8)

## Selection

### TS= "Hospital Formulary" OR "Hospital Formularies" OR "Drug Committee*" OR "Formulary Committee*" OR "Pharmacy Committee*"

### TS= "Essential Drug*"

### TS=Essential Medicine*

### (#11) NOT (#12)

### (#12) OR (#13)

## Insurance and Financing

### TS= "Drug Cost*" OR "Pharmacoeconomic*" OR "Pharmaceutical Economic*" OR "Pharmaceutic Fee*" OR "Pharmacy Fee*" OR "Pharmaceutical Fee*"

### (TS= "Rate Setting* and Review*" OR "Review and Rate Setting*" OR "Review Rate Setting*") AND (#2)

### TI= (reference OR referencing AND (price OR prices OR pricing)) OR (maximum AND (price OR prices OR pricing)) OR (internal OR external AND (price OR prices OR pricing))

### (TS="Health Insurance Reimbursement*" OR "Third Party Payment*" OR "Third-Party Payment*" OR "Third Party Payer*" OR "Third-Party Payer*" OR "Reimbursement Mechanism*" OR "Cost Sharing" OR "Cost-Sharing Insurance" OR "Cost Sharing Insurance") AND (#4)

### (TS=copay OR copays OR copayment OR "co pay" OR "co payment" OR "co payments" OR "fees") AND (#4)

### (#18) NOT (#19)

### (#19) OR (#20)

## Intellectual Property

### (TS="Patents as Topics " OR Trademark*) AND (#1)

### TS= "Intellectual Property Right*" AND "Trade-Related Aspect*"

## Medicines marketing policies

### (TS="Social Marketing" OR "Advertising as Topic*" OR "Advertisement as Topic*") AND (#4)

## Medicines information

### TS= "Drug Industry" OR "Pharmaceutical Industry" OR "Pharmaceutic Industries" OR "Pharmaceutic Industry" OR "Drug Industries" OR "Pharmaceutical Industries" OR "Drug Information Service*"

## Prescribing and Use

### TS="Drug Prescription*"

### (TS="Practice Guidelines as Topic " OR "Best Practice*") AND (#3)

### (TS="Generic Drugs" OR "Nonproprietary Drugs") AND (TS=substitute OR substitution OR substitutions)

### TS="Drug Utilization*"

### (TS="rational use*") AND (TS=pharmaceutical* OR medicine*)

## Medicine Reform/Policy

### (TS="Health Policy" OR "Health Policies" OR "National Health Policy" OR "National Health Policies") AND (#3)

### TS="Pharmaceutical Service*" OR "Pharmaceutic Service*" OR "Pharmacy Service*" OR "Pharmaceutical Care*"

## Medicines access

### (TS= "Health Services Accessibility" OR "Availability of Health Services" OR "Health Services Availability" OR "Access to Health Care" OR "Accessibility of Health Services" OR "Health Services Geographic Accessibility" OR "Program Accessibility" OR "Contraceptive Availability")AND (#3)

## Medicines supply management

### TS="Prescription Drug*" OR "Nonprescription Drug*" OR "OTC Drug*" OR "Over-the-Counter Drug*" OR "Over the Counter Drug*" OR "Patent Medicine*" OR "Non-Prescription Drug*" OR "Non Prescription Drug*" OR "Drug Storage*"

Medicines (All Categories)

### (#5) OR (#9) OR (#10) OR (#14) OR (#15) OR (#16) OR (#17) OR (#21) OR (#22) OR (#23) OR (#24) OR (#25) OR (#26) OR (#27) OR (#28) OR (#29) OR (#30) OR (#31) OR (#32) OR (#33) OR (#34)

### TS="Drug Abuse" OR "Drug Possession" OR "narcotic abuse" OR "narcotic use" OR narcotics OR "substance abuse" OR poison OR poisoning OR venom OR "substance use" OR cocaine OR heroin OR marijauna OR pot OR "salvia divinorum"

### (#35) NOT (#36)

# Health Financing

## Payment mechanisms

### TS="Fee-for-Service Plan*" OR "Fees for Service*" OR "Fee for Service*" OR "Fee-for-Service Reimbursement*" OR "Medical Fee-for-Service*" OR "Physician Incentive Plan*" OR "Employee Incentive Plan*" OR "Reimbursement Mechanism*" OR "Incentive Reimbursement*" OR "Pay for Performance" OR "Prepaid Health Plan*" OR "Prepaid Group Practice*" OR "Capitation Fee*" OR "Salarie* and Fringe Benefit*" OR "Wage*" OR "Salaries" OR "Salary" OR "Paternity Benefit*" OR "Pay Equity" OR "Pay Equities" OR "Fringe Benefit*" OR "Organized Financing" OR "Grant*" OR "Community Financing" OR "Fee* and Charge*" OR "Charge*" OR "Medical Fee*"

## Health insurance

### TS="Health Insurance" OR "Voluntary Health Insurance" OR "Group Health Insurance" OR "Insurance Pools" OR "Insurance Pool" OR "Health Alliances" OR "Health Alliance" OR "Insurance Coverage" OR "Insurance Status"

## Resource Allocation

### TS="Resource Allocation" OR "Resource Allocations" OR "Allocation of Resources" OR "Resources Allocation" OR "Health Care Rationing" OR "Healthcare Rationing" OR "cost-benefit analysis" OR "Cost-Benefit Analyses" OR "Cost Benefit Analysis" OR "Cost Benefit Analyses" OR "Cost Effectiveness" OR "Cost-Benefit Data" OR "Cost Benefit Data" OR "Cost* Benefit*" OR "Benefits and Costs" OR "Costs and Benefits"

Health Financing (All Categories)

#1 OR #2 OR #3

# Governance

## Government regulation and legislation

### TS="Government Regulation" OR "Government Regulations" OR "Government Regulation and Oversight" OR "Legislation as Topic" OR "Laws and Statutes" OR "Statutes and Laws" OR "Health Legislation" OR "Legislation, Health" OR "Population Law" OR "Population Laws" OR "Constitutional Amendments" OR "Constitutional Amendment" OR "Model Legislation" OR "Medical Legislation" OR "Drug Laws" OR "Drug Law" OR "Drug Legislation" OR "Narcotic Laws" OR "Narcotic Law" OR "Hospital Legislation" OR "Hospital Legislations" OR "Pharmacy Legislation" OR "Pharmacy Legislations" OR "Dental Legislation" OR "Nursing Legislation" OR "Nursing Legislations" OR "Facility Regulation and Control" OR "Facility Regulation" OR "Facility Regulations" OR "Facility Control" OR "Facility Controls" OR "Legal Liability" OR "Legal Liabilities" OR "Torts" OR "Tort" OR "Personal Liability" OR "Personal Liabilities" OR "Professional Liability" OR "Professional Liabilities" OR "Institutional Liability" OR "Institutional Liabilities" OR "Medical Liability" OR "Medical Liabilities"

## Licensing and accreditation

### (TS="Health Personnel" OR "Health Care Providers" OR "Health Care Provider" OR "Healthcare Providers" OR "Healthcare Provider" OR "Fieldworkers" OR "Fieldworker" OR "Field Workers" OR "Field Worker") AND (TS= "Licensures" OR "Institutional Personnel Licensure" OR "Institutional Personnel Licensures" OR "Permits" OR "Permit" OR "Licenses" OR "License" OR "Licensing" OR "Credentialing" OR "Accreditation" OR "Accreditations")

## Professional authority and roles

### TS= "Organizational Affiliation" OR "Organizational Affiliations" OR "Staff Development" OR "Human Resources Development" OR "Resources Development, Human" OR "Employee Cross-Training" OR "Professional Role" OR "Professional Roles" OR "Practice Guidelines" OR "Best Practices" OR "Best Practice" OR "Professional-Patient Relations" OR "Professional Patient Relations" OR "Professional-Patient Relation" OR "Professional Patient Relationship" OR "Professional Patient Relationships" OR "Contacting Clients" OR "Contacting Client" OR "Dentist-Patient Relations" OR "Dentist Patient Relations" OR "Dentist-Patient Relation" OR "Dentist-Patient Relationship" OR "Dentist Patient Relationship" OR "Dentist-Patient Relationships" OR "Nurse-Patient Relations" OR "Nurse-Patient Relation" OR "Nurse Patient Relations" OR "Nurse Patient Relationship" OR "Nurse Patient Relationships" OR "Physician-Patient Relations" OR "Physician-Patient Relation" OR "Physician Patient Relationship" OR "Physician Patient Relationships" OR "Physician Patient Relations" OR "Physician Patient Relation" OR "Doctor Patient Relations" OR "Doctor Patient Relation" OR "Doctor-Patient Relations" OR "Doctor-Patient Relation"

## Audit

### TS= "Management Audit" OR "Management Audits" OR "Operational Audit" OR "Operational Audits" OR "Clinical Audit" OR "Clinical Audits"

## Consumer involvement

### TS= "Consumer Participation" OR "Consumer Involvement" OR "Consumer Involvements" OR "Public Participation" OR "Community Action" OR "Community Actions" OR "Community Participation"

Governance (All Categories)

### (#1 NOT #2) AND (#1 NOT #3) AND (#1 NOT #4) AND (#1 NOT #5)

### (#2 NOT #1) AND (#2 NOT #3) AND (#2 NOT #4) AND (#2 NOT #5)

### (#3 NOT #1) AND (#3 NOT #2) AND (#3 NOT #4) AND (#3 NOT #5)

### (#4 NOT #1) AND (#4 NOT #2) AND (#4 NOT #3) AND (#4 NOT #5)

### (#5 NOT #1) AND (#5 NOT #2) AND (#5 NOT #3) AND (#5 NOT #4)

### #6 OR #7 OR #8 OR #9 OR #10

# Health System

TS = ("Health* System*" OR "Health* Care System*" OR " Health* Policy* and System*" OR " Health* Care Policy* and System*")

# Limit Publication Dates and Database

### Publication Date : 01/01/1900 to 31/12/2012

### Database=SCI-EXPANDED, SSCI, A&HCI

All Limits

#1 AND #2

**Final Search strategy:**

**(ⅠOR Ⅱ OR Ⅲ OR Ⅳ OR Ⅴ OR Ⅵ) AND Ⅶ AND Ⅷ**
